# Gut Feeling: Biomarkers and Biosensors’ Potential in Revolutionizing Inflammatory Bowel Disease (IBD) Diagnosis and Prognosis—A Comprehensive Review

**DOI:** 10.3390/bios15080513

**Published:** 2025-08-07

**Authors:** Beatriz Teixeira, Helena M. R. Gonçalves, Paula Martins-Lopes

**Affiliations:** 1 DNA & RNA Sensing Lab, Department of Genetics and Biotechnology, Universidade de Trás-os-Montes e Alto Douro, 5000-901 Vila Real, Portugal; beatcmagalhaes@gmail.com; 2 BioISI—Instituto de Biosistemas e Ciências Integrativas, Faculdade de Ciências, Universidade de Lisboa, 1749-016 Lisboa, Portugal; 3 LAQV, REQUIMTE, Department of Chemistry and Biochemistry, Faculty of Sciences, University of Porto, 4099-002 Porto, Portugal

**Keywords:** IBD, biomarkers, biosensors, diagnosis, prognosis

## Abstract

Inflammatory Bowel Diseases (IBDs) are complex, multifactorial disorders with no known cure, necessitating lifelong care and often leading to surgical interventions. This ongoing healthcare requirement, coupled with the increased use of biological drugs and rising disease prevalence, significantly increases the financial burden on the healthcare systems. Thus, a number of novel technological approaches have emerged in order to face some of the pivotal questions still associated with IBD. In navigating the intricate landscape of IBD, biosensors act as indispensable allies, bridging the gap between traditional diagnostic methods and the evolving demands of precision medicine. Continuous progress in biosensor technology holds the key to transformative breakthroughs in IBD management, offering more effective and patient-centric healthcare solutions considering the One Health Approach. Here, we will delve into the landscape of biomarkers utilized in the diagnosis, monitoring, and management of IBD. From well-established serological and fecal markers to emerging genetic and epigenetic markers, we will explore the role of these biomarkers in aiding clinical decision-making and predicting treatment response. Additionally, we will discuss the potential of novel biomarkers currently under investigation to further refine disease stratification and personalized therapeutic approaches in IBD. By elucidating the utility of biosensors across the spectrum of IBD care, we aim to highlight their importance as valuable tools in optimizing patient outcomes and reducing healthcare costs.

## 1. Introduction

Inflammatory Bowel Diseases (IBDs) represent multifactorial disorders [1] that can be characterized by repetitive, acute inflammation in the gastrointestinal tract, as a response to common antigenic stimuli, due to immune dysregulation [2,3].

Considered a common disorder, it has a prevalence of 0.1–0.4% worldwide [4,5] and is currently affecting 0.8% of the general western population [5,6,7]. In Portugal, IBD prevalence estimates are currently among the highest in Europe [8]. Moreover, in nations that recently have adopted a more industrialized lifestyle (Africa, South America, and Asia), the incidence of this disorder has steeply increased in the last few decades, echoing the expanding incidence of IBD that emerged in Western countries during the 1990s, due to urbanization and rapid socioeconomic growth [5,9].

Presently, there is no known cure; therefore, IBDs require life-long care and impair the quality of life of the patient, frequently needing surgical intervention [4]. Furthermore, the economic burden of healthcare systems worldwide has been rapidly increasing due to the frequent surgical hospitalizations along with the increased use of biological drugs, and increased disease prevalence, which is accompanied by a rising treatment cost [7]. In fact, the overall healthcare burden of IBD patients, in Europe, has been estimated at 4.5–5.6 billion euros per year [8].

The challenges in IBD are mainly focused on five areas: preclinical human IBD mechanisms [10]; environmental triggers [11]; pragmatic clinical research [12]; precision medicine [13], and novel technologies [14].

**Figure 1:**
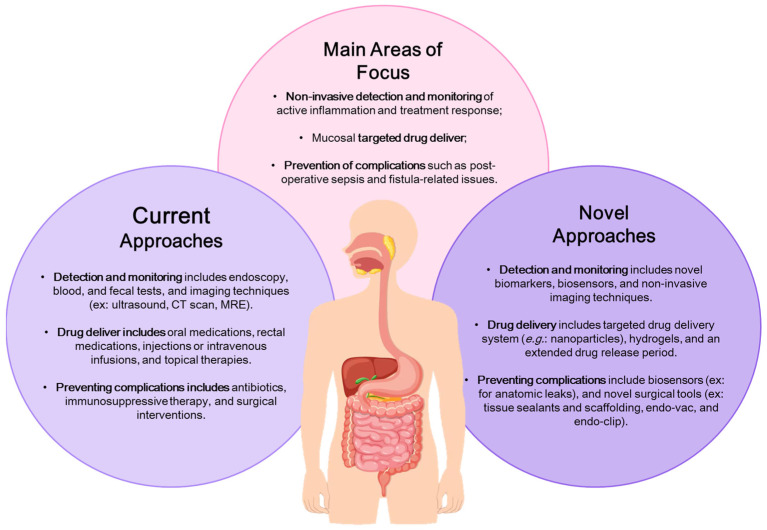
Illustration of the current and novel approaches for addressing unmet clinical needs in Inflammatory Bowel Disease (IBD). Adapted from Dhyani et al. [14].

The novel technologies section is focused on addressing unmet clinical needs in IBD, in three main areas: (1) non-invasive detection and monitoring of active inflammation and assessing treatment response; (2) mucosal targeted drug delivery; and (3) preventing complications, such as post-operative sepsis and fistula-related issues (Figure 1). The proposed solutions involve technologies for localized drug delivery, reducing systematic exposure while enhancing treatment effectiveness, biopolymers, and sealant technologies to support healing after surgery, along with devices to control anastomotic leakage and prevent post-surgical complications and recurrences, and lastly, the creation of advanced imaging methods and biosensors capable of non-invasively or minimally invasively detecting pro-inflammatory signals for monitoring disease activity and treatment responses [14]. The development of biosensors is a crucial alternative to current diagnostic techniques, overcoming logical constraints and providing accurate diagnoses, prognoses, and enabling timely intervention with personalized treatment strategies.

## 2. Inflammatory Bowel Diseases

The two major forms of IBD are ulcerative colitis (UC) (MIM 191390) and Crohn’s disease (CD) (MIM 266600) [2,3,15,16]. Despite their similarities, they are distinct diseases.

UC is caused by diffuse mucosal inflammation affecting primarily the rectum and adjacent colonic tissue, whereas CD is a transmural inflammatory granulomatous disease that can affect any part of the gastrointestinal tract, from the mouth to the anus, affecting mostly the terminal ileum [2,3,15].

Currently, the Inflammatory Bowel Disease’s etiology is unknown. However, research suggests the involvement of genetic and epigenetic susceptibility traits, dysbiosis, environmental factors, long-term colonization/chronic exposure with specific pathobionts, and immune response [1,2,17]. Indeed, not only do IBDs have an unknown origin, but they also have a variable clinical presentation [18]. An IBD patient is very likely to experience a lifetime of debilitating physical symptoms (e.g., diarrhea, rectal bleeding, vomiting, anorexia, and lethargy), leading to impaired psychosocial well-being, which adversely affects academic achievements, employment, relationships, and sexual health [19]. Additionally, about 25% to 40% of individuals with IBD experience extraintestinal symptoms, such as arthritis, axial spondyloarthritis, uveitis, erythema nodosum, and primary sclerosing cholangitis [18,20].

According to Huang et al. [21] and Chen et al. [22], approximately 32.9% of individuals with CD become disabled as a result of the incapacitating symptomology that they produce. In the case of UC patients, 5–15% may require colectomy. In comparison, a substantial 80% of CD patients will undergo at least one surgical intervention throughout their lifetime, which significantly affects their overall quality of life [22,23].

Thus, while inflammatory bowel diseases, including CD and UC, are well characterized by chronic inflammation of the gastrointestinal tract, their clinical presentation remains highly variable. This complexity poses significant challenges in both identifying the precise nature of the disease and predicting its future course. As a result, attention has increasingly shifted toward addressing the diagnostic ambiguities and prognostic uncertainties that often accompany IBD. Understanding these issues is crucial for optimizing patient care and tailoring treatment strategies, making the exploration of diagnostic and prognostic problems a critical next step in the study of IBD.

## 3. Diagnostic and Prognostic Problems in Inflammatory Bowel Diseases

Nowadays, there is no single reference standard for the diagnosis of Crohn’s disease or ulcerative colitis. Thus, the diagnosis of IBDs can be determined by a combination of clinical, biochemical, stool, endoscopic, cross-sectional imaging, and histological tests [24].

Due to the immense variety of symptoms these patients can present, it can be challenging for physicians to promptly diagnose individuals with IBD [18,25]. Certain symptoms can also be mistaken for other illnesses, particularly irritable bowel syndrome (IBS) or hemorrhoids, which can delay diagnosis even further [18,25,26]. Interestingly, one in six people with IBD is misdiagnosed with IBS [26,27], whereas one in seven people with UC is misdiagnosed with hemorrhoids [26,28], which leads to a postponement in the referral to a specialist, and consequently, delay the treatment that can prevent disease progression [26]. Delaying diagnosis might cause adverse effects on clinical outcomes, such as an increased need for further surgical intervention and an inadequate response to medical therapy [18,29,30]. According to recent literature, it can take anywhere from two months to eight years for a person to be diagnosed with IBD [18,25,31]. This reinforces the fact that better and accurate diagnostic methodologies are a pressing need for IBD.

In 2022, a group of researchers conducted a study, in Portugal, in which they evaluated the IBD patient journey, using patient-reported experiences and unmet meets, from symptoms onset to diagnosis, therapy, and follow-up [32]. According to the authors, in a pool of 406 IBD patients, 38% reported a period of more than 1 year to receive a conclusive diagnosis, 49% stated that physicians undervalued their early symptoms, 43% were misdiagnosed initially, and 46% did not receive all the pertinent information regarding their disease when diagnosed. Even though both CD and UC can present extraintestinal occurrences, only 33% of IBD patients reported having multidisciplinary care at the main hospital of follow-up, while 54% were forced to seek extra medical care outside of their primary care healthcare facility, at their own cost. This lack of multifactorial evaluation and treatment in these diseases is a major drawback that affects not only the patient’s quality of life, but also treatment effectiveness. Ultimately, it results in increased costs for both the patient and the healthcare system.

Additionally, a British cohort carried out a similar study [33]. In this study, in 306 patients, 60% were diagnosed within 6 months, 79% within 12 months, and 92% within 24 months of the onset of symptoms, with 4.3 months being the median time to diagnosis. The authors also noticed that patients with Cronh’s disease had a longer time to diagnosis (7.6 months), compared with ulcerative colitis (3.3 months) and other unclassified IBD (3.9 months). The results seem to agree with the ones obtained by an Italian cohort (diagnosis delay being 3.0 months, higher in CD (7.1 months) than in UC (2.0 months)) [28].

Cross et al. [18] conducted a systematic review to understand the extent of diagnostic delay of IBD; medium values from eight articles were analyzed for IBD in general, and it ranged from 2.0 to 13.0 months, with three-quarters of these reporting a delay between 2 and 5.3 months. Similarly, the diagnostic delay was greater for CD: in 23 studies, it ranged from 2.0 to 26.4 months, with three-quarters reporting a delay of 2.0–12.0 months. For UC, in 16 studies, diagnostic delay ranged from 2.0 to 55.2 months, with three-quarters reporting a delay between 2.0 and 6.0 months.

Patients with IBD encounter several obstacles during their care: delays in early diagnosis, access to early treatment, and gaps in multidisciplinary integrated follow-up. All of these are caused by gaps between patients, general practitioners, and a lack of awareness among the general population [32]. Moreover, this gap is also noticed between researchers and clinicians. The lack of contact between these groups leads to an inefficient use of clinical research. The objective of One Health is to provide equal healthcare to all and, in order to do so, it is of the outmost relevance to decrease the gap between researchers and practical clinicians. This is the only way to ensure that precision medicine evolves through the development of tools that clinicians need to provide better care and support a quicker diagnosis. A prompt and differential diagnosis of IBD is crucial for a favorable prognosis and proper treatment responses [34]. Moreover, proper monitoring plays a pivotal role in detecting disease relapse and administering timely treatments.

At the moment, the evaluation of disease activity and treatment response in both adults and children heavily depends on clinical scoring systems like the Crohn’s Disease Activity Index (CDAI). This index is derived from patient-reported symptoms and other variables documented over 7 days [35,36]. The Harvey–Bradshaw Index, also used for UC, is a more concise clinical assessment tool primarily relying on the symptoms reported by the patient at the time of evaluation [36,37]. There are other indices, such as the Simple Index, Mayo Index, Simple Clinical Colitis Activity Index, Oxford Index, van Hees Index, and Cape Town Index, although the CDAI, the Harvey–Bradshaw, and Colitis Activity Index (CAI) are the most used and widespread ones [14,36,38]. Yet, these measures have constraints, including their considerable subjectivity and the frequent disparity between patient-reported symptoms and objective indicators of disease activity, such as endoscopic inflammation and histopathological findings, which have a stronger correlation with long-term outcomes [14].

Serum and fecal (especially) biomarkers are highly valuable in a diagnostic framework when it comes to assisting in the patient selection process for an invasive diagnostic evaluation, such as endoscopy [39].

Nonetheless, endoscopy and histological examinations are time-consuming, expensive, and possess the risk of perforation [22]. The quality of the results obtained by endoscopic approaches is operator- and proper patient-preparation-dependent, and in some instances, they are not sensitive enough to assess clinical severity [40]. Plus, endoscopic imaging is limited to the superficial mucosal layers of the intestine, providing no information regarding muscle thickness, lumen diameter, or inflammatory damage to deeper layers. Moreover, the intricacy of intestinal anatomy, with its multiple loops and folds, and the distance needed to be covered hinders small intestine analysis, especially between the ligament of Treitz and the ileocecal valve, limits even more endoscopy imaging [40,41].

New, less invasive imaging techniques for IBD diagnosis have emerged in recent years, including video capsule endoscopy (VCE), confocal laser endomicroscopy (CLE), and single-/double-ballon assisted endoscopy (SBE, DBE, respectively) [42] (Table 1). However, these approaches also have their disadvantages. With VCE, there is the risk of capsule retention [43], CLE is not ideal for an initial evaluation due to its technical complexity and time-consuming preparation [44,45], and SBE/DBE possess the risk of perforation and bleeding, and they are considered somewhat still as an invasive, expensive procedure that is not recommended as a first-line diagnostic tool in IBD [46,47].

There has been a growing interest in radiological imaging, due to being even less invasive. The most common imaging techniques used for IBD diagnosis and monitoring are currently ultrasound (US), computed tomography (CT), and Magnetic Resonance Enterography (MRE) [24] (Table 1). Conventional ultrasound, while cost-effective, radiation-free, and performed in real-time, is heavily dependent on the operator, which means the quality and the interpretation of results are highly variable, and are limited in evaluating the entire length of the bowel, potentially missing extra-luminal complications due to acoustic attenuation from air within the bowel lumen [14]. CT has constrictions in detecting mild mucosal inflammation and it cannot be used frequently, because of radiation exposure [14]. Recently, MRE has emerged as the primary imaging method for assessing IBDs in patients; however, regulatory agencies have not officially endorsed MRE as a validated disease activity index in clinical trials [14]. Overall, even though these new techniques can overcome some difficulties in diagnosing IBDs, it is still necessary to address various limitations associated with them, such as their relative invasiveness, the need for skilled professionals, use of advanced equipment, their substantial expenses, use of ionizing radiation, patient discomfort, and the inability of some to evaluate the entire gastrointestinal tract comprehensively [14,42]. Moreover, the increasing incidence of these diseases and the subsequent increase in the number of available drug treatments creates a higher demand for imaging methods that can offer validated, early, and frequent assessment of treatment responses [14].

Considering the numerous complications associated with the existing methods in diagnosing IBDs and with their prognosis, there is a pressing need to advance the development of new non-/minimally invasive technologies (e.g.,: biosensors), to promptly diagnose, and monitor Inflammatory Bowel Diseases, besides assessing treatment responses.

**Table 1:** Current methods of diagnosis and their respective advantages and disadvantages.

Methods of Diagnosis	Advantages	Disadvantages	References
**Endoscopy**	-Biopsy collection; -Assess disease severity; -Therapeutic intervations;	-Invasive procedure; -Risk of complications; -Cost and availability; -Results are operator- and proper patient-preparation-dependent; -Limited to the superficial mucosal layers of the intestine; -Cannot image all parts of the intestine, due to its anatomy	[22,40,41]
**Video Capsule endoscopy (VCE)**	-Less invasive than an endoscopy; -Images of the whole bowel	-Risk of capsule retention; -Even more cost-effective; -Cannot be used to perform biopsies; -Lack of therapeutic capabilities	[42,47]
**Confocal laser endomicroscopy (CLE)**	-Used in vivo, allows to obtain living tissue images during colonoscopy; -Faster diagnosis	-Technical complexity; -Time-consuming preparation -Risk of complications; -Cost and availability;	[42,44,45]
**Single-/Double-ballon assisted endoscopy (SBE, DBE, respectively)**	-Images of small bowel areas a standard endoscopy cannot reach	-More invasive than a standard endoscopy; -Technical complexity; -Specialized training and expertise by endoscopists is needed; -Cost and availability	[42,47]
**Ultrasound**	-Cost-effective; -Radiation-free; -Performed in real-time	-Heavily dependent on the operator; -Limited in evaluating the entire length of the bowel	[14]
**Computed tomography (CT)**	-Can assess bowel wall thickening, mesenteric edema, lymphadenopathy, inflammatory masses and abcesses (Cross-sectional imaging capability)	-Limited in detecting subtle mucosal inflammation; -Radiation exposure; -Patient discomfort; -Cost and technical complexity.	[14]
**Magnetic Resonance Enterography (MRE)**	-Cross-sectional imaging capability; -Lack of ionizing radiation	-Cost and technical complexity; -Patient discomfort	[14]

## 4. Biomarkers in Inflammatory Bowel Diseases

The need to tailor treatments for each individual and adapt them as the disease progresses is essential. Some of the factors that need to be considered are the severity of the condition, the extent of the lesion, disease characteristics, and how the patient responds to medications. Vigilant monitoring of treatment responses is essential to gauge their efficacy and prevent possible complications. Indeed, research on individuals with CD reported that mucosal healing enhances the likelihood of steroid-free remission and decreases the chances of re-hospitalization and need for surgery [22,48,49].

**Figure 2:**
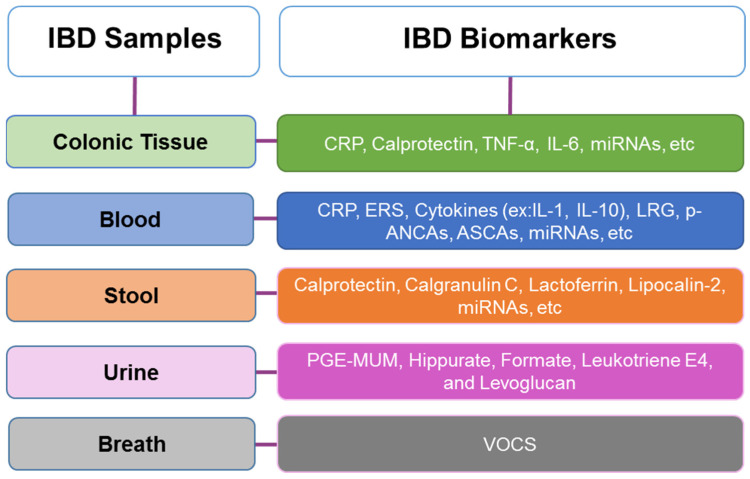
Inflammatory Bowel Disease (IBD) most common samples and associated biomarkers, and where they are present [34,42,51,52,53,54].

An ideal biomarker in IBD should be non-invasive, sensitive, specific to the disease, easy to use, cost-effective, reproducible between laboratories and individuals, able to measure disease activity, capable of monitoring treatment effectiveness, and have a prognostic value [22,50]. Currently, no biomarker combines all these qualities to accurately diagnose IBD, in distinguishing between subtypes, monitoring disease activity, or having a prognostic value. However, some specific biomarkers have been identified in colonic tissue, blood, stool, urine, and even breath (Figure 2). Among these, blood-based biomarkers are the most commonly used, e.g., serological markers and antibodies [22]. When there is a suspicion of IBD, laboratory tests can assist in directing further investigation and aiding in the differential diagnosis [34,39].

### 4.1. Serological Markers

Routine laboratory tests for IBD diagnosis often include serum biomarkers. Not exclusive to IBD, they are widely employed in initial diagnosis since they are easy to use, cost-effective, and have established protocols (Table 2). The most prevalent tests include those for C-reactive protein (CRP) and the erythrocyte sedimentation rate (ESR) [42].

#### 4.1.1. Serological Markers for CRP

CRP is a liver-produced pentameric protein, typically present in the bloodstream at <1 mg/L under normal physiological conditions [42,55]. Its levels can rise during an acute-phase response when pro-inflammatory cytokines like IL-6, tumor necrosis factor α (TNF-α), and IL-1β stimulate its production in the hepatocytes [42,55,56]. Having a consistent half-life of 19 h, CRP plasma concentration is primarily determined by the synthesis rate, thus its levels directly reflect the intensity of pathological stimulation in the body [55]. Nowadays, turbidimetric and nephelometric techniques with CRP enzyme-linked immunosorbent assay (ELISA) kits are employed to detect CRP in real samples [57]. Although robust and affordable, they need advanced equipment and multiple samples, exhibit selective detection, and demand a lengthy process to yield results, being time-consuming and involving multiple steps [57,58]. This protein, when released, goes into the damaged tissue, and triggers a response called complement, which has pro-inflammatory effects and promotes beneficial scavenging functions. This process can worsen tissue damage and can contribute to more advanced disease. Thus, measuring CRP levels in the bloodstream is highly suggested as a biological marker in several diseases, capable of assessing the severity and extent of IBD [55,59]. However, CRP is a poor parameter of inflammation in UC, since about 50% of UC patients have normal CRP levels during a flare-up [34].

The ESR test is a simple and cost-effective way to detect acute inflammation in IBD patients. It measures how quickly erythrocytes settle to the bottom of a vertical column of anticoagulated blood, under the influence of gravity [42,55]. ESR is mainly influenced by two factors: the extent of red blood cell clustering and hematocrit (volume of packed red blood cells). The clustering of the erythrocytes is affected by the proteins present in blood plasma [55,60]. In cases of an active inflammatory response, the erythrocytes will settle at a faster rate, causing increased clustering; this can be used to measure the inflammatory activity caused by IBD [42,55,61].

However, besides being a well-studied diagnostic tool for IBD, these tests are not considered gold-standard due to their lack of specificity and accuracy. CRP offers certain advantages over ESR, i.e., it responds faster to changes in disease activity, has a wider range of abnormal values, and does not vary with age and medication, unlike ESR [42]. Nonetheless, they are not IBD-specific and a positive result may be associated with a non-related inflammatory process.

#### 4.1.2. Serological Markers for LRG

A novel serological biomarker for IBD and rheumatoid arthritis has recently emerged. A leucine-rich alpha 2-glycoprotein (LRG), 50 kD protein, is produced by hepatocytes, neutrophils, macrophages, and intestinal epithelial cells [62]. Research indicates that LRG levels rise in patients with active UC and decrease as the disease enters remission. Notably, increased LRG levels show a stronger association than CRP, with clinical and endoscopic scores in both UC and CD patients with active disease [42,62,63,64,65]. This protein has also been found to predict mucosal healing in patients with UC and CD, even when CRP levels are normal [65]. Thus, this is a non-specific but quite promising biomarker for early IBD diagnostic and crisis follow-up.

**Table 2:** Comparison of serological IBD markers: quantities between healthy cohorts and patients with IBD, detection techniques, advantages, disadvantages, and their diagnostic performance (sensitivity and specificity).

Biomarker	Quantity	Detection Technique	Advantages	Disadvantages	Sensitivity (Se) Specificity (Sp)	Reference
CRP	Normal: <1 mg/L Mild inflammation/Viral infections: 10–40 mg/L; Severe active inflammation/Bacterial infection: 50–200 mg/L	Turbidimetric and nephelometric techniques with CRP enzyme-linked immunosorbent assay (ELISA) kits	Robust and affordable; This biomarker can access the extensibility and severity of the disease	Detection Technique: Advanced equipment; Multiple samples; Exhibit selective detection; Time-consuming and involving multiple steps. Biomarker: Not IBD-specific, low specificity	Se: 77–82% for UC; 83–92% for CD Sp: 32–40% for UC; 70–89% for CD	[50,57,59,65,66,67,68]
Erythrocyte sedimentation rate (ESR)	Normal: Male < 50 years old: ≤15 mm/hr Female < 50 years old: ≤20 mm/hr Male > 50 years old: ≤20 mm/hr Female > 50 years old: ≤30 mm/hr Child: ≤10 mm/hr Abnormal: >20 mm/hr	Measured by various methods: Westergren, Wintrobe, micro-ESR, and automated machines.	Simple and cost-effective way to detect acute inflammation	Detection Technique: Technical factors (amount of blood drawn into the tube, vibrations, temperature, time from specimen collection, the addition of proper anticoagulants, and tube orientation) can affect the results; time-consuming; medication can affect results. Biomarker: Not IBD-specific, low sensitivity and specificity	Se and Sp: Around 78%	[42,69,70]
Leucine-rich alpha 2-glycoprotein (LRG)	Reference values range vary with according to the Assay method (different kits/labs); Sample type (serum vs. EDTA/heparin/citrate plasma); Population (age, health status) Normal: There are no male/female differences in normal LRG values. Serum (Elisa): 19–40 ng/mL. Plasma/Serum: 21–50 µg/mL. Abnormal: ≥10.8 μg/L for UC and ≥13.4 μg/L for CD	Immunoturbidimetric or immunoenzymatic assays (such as Nanopia kit and ELISA kits)	Robust and affordable; Can predict mucosal healing in patients with UC and CD, even when CRP levels are normal	Detection Technique: Advanced equipment; Multiple samples; Exhibit selective detection; Time-consuming and involving multiple steps. Biomarker: Not IBD specific, lack of knowledge about this protein and its correlation with IBD	Se: 87.9–99.3% for UC; 68–96% for CD Sp: 86.2–99.9% for UC; 87–97% for CD	[65,71,72,73]

### 4.2. Serological Antibodies

Although being a well-established diagnostic tool for other immune diseases, in IBD, they are mainly used after a confirmed diagnosis, with limited exploration as a primary diagnostic tool for suspected cases [42]. Two main antibodies, perinuclear anti-neutrophil cytoplasmic antibodies (p-ANCAs) and anti-Saccharomyces cerevisiae antibodies (ASCAs), are commonly studied in IBD research (Table 3). ANCAs are a group of antibodies produced against antigens in the cytoplasm of neutrophils, while ASCAs target mannan and other yeast cell wall components. Both can help to predict UC or CD but have low accuracy and sensitivity [42]. Nevertheless, offering valuable positive or negative predictive values, they can help distinguish between IBD subtypes: p-ANCA+/ASCA− is often found in UC patients, whereas p-ANCA−/ASCA+ is more common in CD patients [42,55,74].

**Table 3:** Comparison of serological antibody IBD markers: quantities between healthy cohorts and patients with IBD, detection techniques, advantages, disadvantages, and their diagnostic performance (sensitivity and specificity).

Biomarker	Detection Technique	Advantages	Disadvantages	Sensitivity (Se) Specificity (Sp)	Reference
Perinuclear anti-neutrophil cytoplasmic antibodies (p-ANCAs)	Indirect immunofluorescence	Can help distinguish between IBD subtypes: p-ANCA+/ASCA− is often found in UC patients, whereas p-ANCA−/ASCA+ is more common in CD patients	**Biomarker:** Not IBD-specific, low sensitivity, low accuracy **Detection Technique**: Complex, time-consuming, background noise, subjective, and expensive	Se: 31–34% Sp: 96–98%	[34,75,76,77]
Anti-*Saccharomyces cerevisiae* antibodies (ASCAs)	ASCA enzyme-linked immunosorbent assay (ELISA), Indirect immunofluorescence	Robust and affordable; can help distinguish between IBD subtypes: pANCA+/ASCA− for UC; pANCA−/ASCA+ for CD	**Detection Technique**: Advanced equipment; Multiple samples; Exhibit selective detection; Time-consuming and involving multiple steps. **Biomarker:** Not IBD-specific, low sensitivity, low accuracy	Se: 38–42% Sp: 91–94%	[34,78,79]

### 4.3. Fecal Biomarkers

Specifically, present in stool samples, the reported fecal biomarkers for IBD primarily include fecal leukocyte proteins, such as calprotectin, calgranulin C, lactoferrin, and lipocalin-2 (Table 4). These biomarkers offer advantages over serological biomarkers, including ease sample accessibility, higher biomarker concentration due to direct contact with the inflamed site, and greater specificity for IBD as they reflect gastrointestinal inflammation, unlike serum biomarkers that are affected by various inflammation types [42,80].

Calprotectin, a calcium-binding protein from the S100 family (S100A8 and S100A9), is expressed by leukocytes and is commonly released during infections and inflammatory processes [39,81]. Found in abundance in neutrophils, eosinophils, and macrophages, calprotectin levels change in various secretory and excretory products when granulocytes and mononuclear phagocytes are activated [42]. Its sensitivity and specificity vary among different assays, and sample collection can be challenging [39,81]. Recently, serum calprotectin has been gaining attention as an IBD biomarker since it can be easily used in routine practice, as it is easier to obtain a sample. Some reports propose that elevated serum calprotectin (SC) levels are associated with intestinal inflammation in both CD [82] and UC [83], making it a potential predictor for IBD diagnosis [39,81].

Another member of the S100 calcium-binding protein family, predominantly expressed by neutrophils, is calgranulin C. This protein is recognized for its pro-inflammatory role, as it binds to RAGE (receptor for advanced glycation end-products) on endothelial cells, mononuclear phagocytes, and lymphocytes, leading to the upregulation of pro-inflammatory cytokines. With a sensitivity of 86% and specificity of 96%, calgranulin C aids in differentiating active IBD from IBS [84].

In turn, lactoferrin is a glycoprotein derived from neutrophils, and serves as an indicator of intestinal inflammation in both IBD and infectious gastroenteritis and it is proven to be one of the most sensitive (94%) for distinguishing IBD from non-IBD [34,85].

Finally, lipocalin-2 (LCN-2), alternatively known as neutrophil gelatinase-associated lipocalin (NGAL) and siderocalin (Scn), is a bacteriostatic protein stored within neutrophil granules [42,53,86]. This protein plays a role in innate immunity by sequestering iron from pathogenic bacteria, thereby limiting their invasion. Highly stable, it exhibits increased expression in colonic biopsies from inflamed regions of patients with IBDs, particularly UC. Thus, serum LCN-2 has been established as a valuable biomarker for UC patients. It is also commonly utilized as a fecal biomarker for acute inflammation in animal models of UC, suggesting its potential utility as a fecal biomarker for human UC. Upregulation of LCN-2 is thought to be induced by IL-22 and IL-17A cytokines [42,87].

**Table 4:** Comparison of faecal IBD markers: quantities between healthy cohorts and patients with IBD, detection techniques, advantages, disadvantages, and their diagnostic performance (sensitivity and specificity).

Biomarker	Quantity	Detection Technique	Advantages	Disadvantages	Sensitivity (Se) Specificity (Sp)	Reference
Calprotectin	Normal: Not yet established (possibly around 150–250 μg/g) Abnormal: >250 μg/g	Turbidimetry, CLIA and ELISA assays	Stable biomarker and resistant to degradation. Detection techniques are robust and affordable	Its sensitivity and specificity vary among different assays, and sample collection can be challenging. Not IBD-specific. Advanced equipment; Time-consuming and involving multiple steps	Se: 88% Sp: 80%	[34,50,88]
Calgranulin C	Abnormal: cut-off of 10 mg/kg	Sandwich enzyme-linked immunosorbent assay (ELISA)	Stable, resist degradation. Detection techniques are robust and affordable. Possible to discriminate IBD with/without mucosal lesions and IBS.	Detection technique involves advanced equipment, is time-consuming and involves multiple steps	Se: 96% Sp: 92%	[89,90,91]
Lactoferrin	Normal: <7.25 μg/g Abnormal: >7.25 μg/g	Quantitative ELISA	Stable, and its extracellular release is the most efficient. Resistant to degradation	Detection technique involves advanced equipment, is time-consuming, and involves multiple steps	Se: 82% Sp: 95%	[34,92,93]
Lipocalin-2 (LCN-2)	Abnormal: >6700 ng/g	Sandwich enzyme-linked immunosorbent assay (ELISA)	Valuable marker for UC, helping distinguish between IBD subtypes	Detection technique involves advanced equipment, is time-consuming, and involves multiple steps	Se: 85.7% for CD and 82% for UC Sp: 45.5% and 80% for UC	[86,94]

## 5. Genetics and Epigenetics Behind Inflammatory Bowel Diseases

The significance of genetic and epigenetic elements in both Crohn’s disease and ulcerative colitis has been widely acknowledged. One of the highest risk factors for developing IBDs is having a family member with the condition, which is more significant among siblings than among offspring. There is a higher risk for a sibling of an individual with CD, 13 to 36 times higher incidence, while for UC, the span is of 7 to 17 times when compared to the general population [95,96]. Additionally, the heritability coefficients for siblings of individuals IBD probands—those who are the first affected in a family—show that genetic factors play a significant role in the development of the disease. For Crohn’s disease (CD), these coefficients range from 25 to 42. For ulcerative colitis (UC), the coefficients range from 4 to 15. When considering pooled twin studies, the heritability estimates for CD and UC are 0.75 and 0.67, respectively [96,97].

Over the past two decades, genome-wide association studies (GWASs) have significantly expanded our understanding of the genetics of IBDs, having identified more than 200 genes associated to the risk of presenting the diseases [98].

Most of the identified Single-Nucleotide Polymorphisms (SNPs) exhibit associations with both UC and CD and are summarized in Table 5, suggesting similar disease susceptibility and common signaling pathways [98,99]. The increased understanding of disease mechanisms related to intestinal homeostasis allowed a better identification of mechanistic pathways. This notably includes regulations in epithelial and barrier function, adaptive immunity, host–pathogen interactions involving immune responses, and autophagy [98]. Moreover, over 60% of the susceptibility loci are linked to other immune-mediated diseases, such as psoriasis, celiac disease, and ankylosing spondylitis [98]. To summarize, the strongest genetic associations have been observed in IBDs for the interleukin-24 receptor (IL-23R) gene, in CD for nucleotide-binding oligomerization domain-containing protein 2/caspase recruitment domain family member 15 (NOD2/CARD15) gene, and in UC for the human leukocyte (HLA) complex [98,100].

Nevertheless, genetic factors only account for a portion of the overall disease variability in IBD. Microbiota and environmental factors may interact with genetic susceptibility in the pathogenesis of IBD. Traditionally, the primary role in the development of IBD has been attributed to the adaptive immune system; however, recent research in genetics and immunology has affirmed the significant involvement of the innate immune system in triggering gut inflammation. New advances in comprehending IBD pathogenesis shed light on crucial disease mechanisms, including the innate and adaptive immunity and interactions between genetic, microbial, and environmental factors [1].

The epigenetic role in IBD has been unravelling over these past years. Epigenetic alterations refer to molecular mechanisms that can adaptively modify gene expression in response to environmental cues, independently of the genetic code. Nowadays, several potential clinical applications of epigenetics in diagnostics and therapeutics are under consideration. In diagnostics, epigenetics hold promise for utilizing biomarkers for diagnostic validation, stratify disease progression, and assess responsiveness to chemotherapy, as well as predict the likelihood of cancer development [1]. In 2000, it was proposed that epigenetics had a significant involvement in the pathogenesis of IBD [101], mainly through histone modification, non-coding RNAs, and DNA methylation [102].

DNA methylation involves the addition of a methyl group to cytosine nucleotides, particularly in the CpG dinucleotide sequence, which constitutes about 1–2% of the genome. Typically, humans have low transcriptional activity; however, elevated DNA methylation can significantly alter the transcriptional activity and gene expression, influencing disease risk and progression. Numerous studies have reported distinct mucosal methylation changes in various genes among patients with IBD compared to healthy individuals, often revealing significant differences between CD and UC [100]. Table 6 highlights the role of DNA methylation in the context of IBD.

Some studies have demonstrated that the combination of multiple markers can achieve an accuracy of 70–80% in distinguishing between IBD and control groups [100,103,104]. Nevertheless, limitations arise from different methylation profiles between distinct cell types and sites, as well as technical constraints and the associated costs of the technology [102].

MicroRNAs (miRNA) are a set of non-coding RNAs responsible for RNA silencing and gene expression. These short nucleotide sequences can regulate gene expression at both transcriptional and post-transcriptional levels, affecting various pathways. In the context of IBD, they may intervene in T-cell differentiation, TH17 signaling pathway, and autophagy. Additionally, they may exhibit distinct patterns of differentiation between CD and UC [100]. An interesting fact is that these miRNAs can be detected in fecal samples, and alterations in miRNA expression were observed in patients with IBD, with the highest expression of miR-155 and miR-223 in testing and validation cohorts (Table 7). miR-21, miR-155 and miR-223, display significant levels, which suggest their potential utility as IBD biomarkers [34]. Table 7 summarizes how miRNAs can be involved in IBD. This paves the way for the development of new and cost-effective diagnostic kits that can detect these miRNAs in fecal samples, which is a less invasive detection method.

## 6. Biosensors and Their Clinical Applications in IBD

The global incidence of IBD has been increasing steadily [40,105,106]. Besides the technological advances in the medical field, it is urgent to develop better and faster diagnostic methods [107]. IBDs, encompassing Crohn’s disease and ulcerative colitis, are chronic, relapsing inflammatory conditions of the gastrointestinal tract with a complex, multifactorial etiology involving genetic susceptibility, environmental triggers, dysbiosis of the gut microbiota, and dysregulated immune responses [40]. This intricate pathophysiology leads to considerable heterogeneity in clinical presentation and disease progression, posing significant challenges for accurate diagnosis, disease monitoring, and therapeutic decision-making [105,106]. Current diagnostic tools—such as endoscopy, imaging techniques, and serum or fecal biomarkers—while indispensable, are often invasive, costly, time-consuming, and limited by their inability to capture dynamic disease activity in real-time. Furthermore, these traditional approaches are largely clinic-centered and episodic, failing to reflect the continuous and fluctuating nature of IBD [105,106]. In response to these limitations, biosensor technologies are emerging as a transformative solution, offering minimally invasive, rapid, and continuous monitoring of physiological and molecular markers relevant to intestinal inflammation [107,108]. By enabling real-time tracking of disease activity, biosensors have the potential to support more personalized and responsive care strategies. When integrated into a patient-centric model, such technologies empower individuals to participate actively in disease management, improve communication with healthcare providers, and facilitate earlier intervention during disease flares. Thus, biosensors represent a promising frontier in addressing current diagnostic gaps and advancing precision medicine in the management of IBD [107,109,110,111].

Non-invasive sensing technology that continuously measures various clinical parameters related to IBD, such as intestinal inflammation, mucosal healing, microbiome changes, and immune system dysregulation, offers valuable potential for early detection of disease activity in asymptomatic patients and monitoring therapy responses [14]. Therefore, it should be able to detect signals linked to processes closely associated with intestinal inflammation, while also detecting an active ‘flare-up’ before symptoms occur, and that is able to distinguish between symptoms caused by inflammation and those occurring in their absence [14].

These analytical devices, ideally small, combine physicochemical and biological components to produce a quantifiable signal, intended to detect a biological analyte. The binding of different biorecognition elements (e.g.,: antibodies, nucleic acids, aptamers, and enzymes) to their complementary counterparts causes a physicochemical change in the sample’s properties, which is detected by a transducer that converts the information into a physical quantity [107,110,111].

The ideal biosensor should be non-invasive, or minimally invasive, offering real-time or near-real-time, continuous or periodic, data sampling and reporting. Their use should enable routine monitoring of the disease in daily life, without requiring visits to healthcare facilities. Must be practical, patient-friendly, and have a high potential for rapid approval in the consumer market with cost-effective production [14].

A potential sensing technology for monitoring IBD is the intestinal gas capsule, capable of detecting changes in the composition of luminal gas, specifically variations in hydrogen and hydrogen sulphide that may be linked to the worsening of IBD [14,112]. Ongoing research involves the use of engineered commensal bacteria and yeast designed to detect different inflammation mediators. Envisioning a “sense and respond” version, this would involve bacteria not only sensing inflammation, but also delivering an anti-inflammatory signal in response [14].

Recently, a group of researchers developed a study in which they measure, characterize, and validate calprotectin expression in human sweat [39]. This study marks the pioneering demonstration of CP detection in sweat using a wearable sweat sensor device. The sensor exhibited a broad dynamic range of 0.1–10 μg/mL and a limit of detection (LOD) of 0.1 μg/mL. Notably, the sensor displayed CP specificity, showing minimal to no response to non-specific inflammatory markers, such as C-reactive protein and interleukin-6. This is highly relevant since human subject studies revealed elevated basal CP levels in IBD subjects compared to a healthy cohort. Moreover, a threefold increase in CP levels was observed during flare-ups in IBD subjects, with a mean concentration of ~1300 ng/mL. These preclinical findings of elevated CP levels hold significant promise for clinicians, as they offer a non-invasive and non-intrusive method for tracking flare-ups in IBD patients, potentially enhancing disease management. Hence, sensors targeting CP present great potential in precision medicine.

CRP stands out as the extensively studied biomarker in sensing platforms. Yang and co-workers [57] designed a biosensor platform based on CRP-affinity peptides for the precise quantification of CRP in blood samples from Crohn’s disease patients. The peptide-based biosensor was created by immobilizing the CRP-affinity peptide onto the surface of a gold nanocomposite, more specifically AuNPs@BP@PDA. The quantitative analysis was achieved by measuring the inhibition efficiency of the Square Wave Voltammetry (SWV) peak current on the sensor. The constructed biosensor demonstrated high sensitivity in quantifying CRP, within the range of 0–0.036 μg/mL, with a low LOD of 0.7 ng/mL. These results highlight the effectiveness of the detection system, combining a unique affinity peptide with the AuNPs@BP@PDA nanocomposite, for monitoring CRP in real samples from Crohn’s disease patients.

Jagannath et al. [113] further developed their wearable sensor for CP detection and developed a multimode SWEATSENSER designed for the non-invasive and continuous analysis of C-reactive protein and interleukin-1β. The sensor exhibited the capability to detect interleukin-1β and C-reactive protein in sweat over a dynamic range spanning 3 log orders. This research marks the first compelling evidence of identifying multimode cytokines and inflammatory markers in passively expressed eccrine sweat using a portable and wearable form factor, offering potential advancements in the management of IBD.

Another interesting sensing system was developed by Thangamuthu et al. [114]. These researchers devised an economical, straightforward, and label-free electrochemical immunoassay for quantifying CRP in a small serum sample drop. The immunosensor strip used here is composed of a screen-printed carbon electrode (SPE) modified with anti-CRP-functionalized gold nanoparticles (AuNPs). The assay capitalizes on the reduction in oxidation current of the redox indicator Fe^3+^/Fe^2+^, stemming from the immunoreaction between CRP and anti-CRP. Under optimal conditions, this immunoassay can measure CRP within a linear range of 0.4–200 nM (0.047–23.6 μg/mL), exhibiting a LOD of 0.15 nM (17 ng/mL, S/N = 3) and a sensitivity of 90.7 nA/nM. Additionally, the method demonstrates good reproducibility and storage stability [114].

Moreover, an expeditious immunoassay was developed to determine adalimumab—a fully human monoclonal antibody that specifically targets tumor necrosis factor-alpha (TNF-α) to initiate and sustain clinical remission in individuals with moderate-to-severe cases of CD and UC—concentration. This assay uses an optical biosensor, and had strong correlation with ELISA results, which presents a promise that this new biosensor could emerge as an innovative and cost-effective point-of-care diagnostic tool for measuring adalimumab in hospital settings or infusion centers [58]. This allows for a more precise, personalized therapy, permitting patients to enter remission earlier.

Significant strides have been made in the progress of treatments for IBD, primarily due to advancements in nanobiotechnology. In this context, nanoparticles (NPs) equipped with diverse engineered properties suitable for biological applications have emerged as valuable instruments, revolutionizing the fields of disease diagnosis, treatment, and theranostics [115]. Nevertheless, nanomaterials (NMs) can also be used for IBD diagnosis. Table 8 illustrates recent biosensing investigations, showcasing various Inflammatory Bowel Disease (IBD) biomarkers and their respective limits of detection (LODs).

Additionally, Shepherd et al. [134] devised a stool analysis method employing headspace gas chromatography coupled with chemiresistive metal oxide gas sensors. They utilized artificial neural network software to analyze the temporal variation in resistance data obtained from diverse samples IBD detection. The system has shown capability to distinguish between samples associated with IBS and IBD, achieving a sensitivity of 76% and specificity of 88%. The overall mean predictive precision of the technique was around 76%.

Fang et al. [122] developed a novel DNA/AgNC-cDNA probe for the detection of miRNA using a fluorescence-enhancing (turn-on) strategy. Targeting miR-223, a potential biomarker IBD, they designed a fluorescent probe with partially hybridized DNA/AgNC-cDNA. The cDNA sequence, fully complementary to miR-223, acted as a quencher for the fluorescent DNA/AgNC segment. In the presence of miR-223, which competitively bound to the cDNA, the DNA/AgNC was liberated from the DNA/AgNC-cDNA complex, leading to an increase in fluorescence. This fluorescence sensing system is quite interesting since it relies on fluorescence enhancement rather than quenching, which is often associated with poor results for low analyte concentrations.

On top of that, Gholami et al. [124] engineered a highly sensitive nanosensor for Surface-Enhanced Raman Spectroscopy (SERS) employing a gold-coated copper oxide nanomaterial. This nanosensor demonstrated exceptional capability in identifying tumor necrosis factor-alpha (TNF-α) in blood, with an LOD reaching 173 pg/L. The SERS quenching sensor’s accuracy in cytokine measurement was validated through cross-comparison with the enzyme-linked immunosorbent assay (ELISA), revealing a substantial 93.39% agreement between the two methodologies.

Despite the great advances in terms of detection tools, there is still much more to be done in order to develop more effective technologies for IBD diagnosis. Indeed, as IBD presents itself as a multifactorial disease, the best way to obtain promising diagnostic tools also needs a multi-stage approach. First stage: it is necessary to better understand the underlying biological mechanisms of IBD and determine the associated specific biomarkers. Moreover, it is very important that these biomarkers are evaluated before, during, and after a crisis in order to best optimize the patient follow-up and treatment prognosis, requesting a complete validation procedure. Second stage: when these biomarkers are determined, it is important to develop specific biosensors that can detect them in different biological matrixes, e.g., sweat, serum, and feces. Third stage: the developed biosensors need to be able to be miniaturized (or to be portable), easy to operate, and cost-effective for point-of-care application. IBD precision medicine can only be obtained using a multifactorial path, where different areas work in straight collaboration.

Factors such as cost, scalability, and ease of operation are key to determining the viability of the designed sensors to reach the market and become valuable tools in detecting and managing of IBD. Taking into consideration the sensors described within this review, a comparison between these critical issues is presented in Table 9.

## 7. Future Perspectives

In recent years, the management and diagnosis of inflammatory bowel diseases (IBDs), including Crohn’s disease and ulcerative colitis, have entered a new era driven by advances in **materials science**, **microfluidics**, and **artificial intelligence (AI)**. These interdisciplinary innovations are reshaping how clinicians detect, monitor, and treat chronic intestinal inflammation and can be divided into smart materials for responsive sensing and drug delivery, lab-on-chip and organ-on-chip systems, artificial intelligence, and machine learning integration.

Smart materials—especially responsive polymers, nanocomposites, and adaptive hydrogels—are being increasingly employed in biosensing and therapeutic systems for IBD. These materials are engineered to respond to specific biomarkers such as calprotectin, CRP, or inflammatory cytokines (e.g., IL-6, TNF-α), enabling real-time sensing and targeted therapeutic release. A great example of this are the wearable platforms that use functionalized polymers or graphene-based conductors that can detect biomolecules in sweat or interstitial fluid [128,129]. These materials enhance sensitivity and flexibility, making them ideal for continuous, non-invasive monitoring. Stimuli-responsive drug delivery systems are also being developed, releasing anti-inflammatory agents only when inflammatory signals reach pathological levels—potentially minimizing side effects and improving efficacy.

Lab-on-chip devices, particularly microfluidic platforms, have become valuable tools for both diagnostics and disease modeling in IBD. Droplet microfluidics and digital ELISA systems now enable high-sensitivity biomarker detection from minimal samples, supporting rapid, decentralized testing [131]. Simultaneously, gut-on-chip platforms—microfluidic models mimicking the intestinal environment—are being used to study host–microbiota interactions, barrier dysfunction, and drug responses under dynamic flow and oxygen gradients. These chips are increasingly integrated with sensors and imaging systems, offering a physiologically relevant alternative to traditional in vitro models.

The fusion of AI and machine learning (ML) with diagnostic and monitoring tools is revolutionizing IBD care. In digital pathology, deep learning algorithms now accurately classify inflammatory severity from histological slides, often surpassing interobserver reliability in clinical assessments. In endoscopy, AI-powered tools are used for real-time grading of mucosal inflammation, enabling consistent disease scoring during colonoscopy [135]. Moreover, AI models are being deployed in lab-on-chip data analysis—for instance, in microfluidic droplet classification, cellular sorting, and real-time signal interpretation. These applications enhance speed, precision, and automation in complex diagnostic pipelines. Predictive models trained on longitudinal patient data can forecast disease flares and treatment response, contributing to precision medicine approaches in IBD.

The convergence of **smart materials**, **miniaturized fluidic platforms**, and **AI-driven analytics** holds great promise for IBD management.

An alternative approach is the development of oral sensors. IBDs are increasingly recognized as disorders of the gut–brain axis, involving systemic immune dysregulation and a disrupted microbial balance, not only in the gut but also in the oral cavity. Emerging research highlights the mouth–gut microbial axis, where dysbiosis in the oral microbiome (e.g., *Prevotella*, *Fusobacterium*, *Porphyromonas*) may reflect or even contribute to intestinal inflammation [136]. In this context, biosensors could be developed to monitor the mouth microbiome. New biosensors could target specific biomarkers, such as shifts in microbial composition (e.g., Fusobacterium nucleatum, Porphyromonas gingivalis, among others), Inflammatory cytokines IL-1β, IL-6, and TNF-α in gingival crevicular fluid (GCF), or saliva and Bacterial 16S rRNA, miRNAs associated with oral inflammation.

Overall, integrated systems capable of sensing, processing, and responding to disease signals autonomously are emerging, laying the groundwork for personalized, real-time disease monitoring and intervention. These technologies are not only advancing diagnostics but also paving the way for closed-loop therapeutic systems—potentially transforming IBD from a reactive to a proactive field of care.

Looking ahead, the successful clinical translation of biosensor technologies in IBD management will require addressing several key challenges. Regulatory approval remains a significant hurdle, as biosensors must meet rigorous standards for safety, efficacy, and data reliability before widespread clinical adoption. Moreover, according to the current EU legislation, these biosensors are required to pass through a Notified Body regulation procedure that is a time-consuming and costly process. Ensuring long-term biocompatibility is another critical concern, particularly for implantable or wearable devices, which must maintain functionality without provoking adverse immune responses or degradation over time. Additionally, for biosensors to be effectively integrated into routine care, seamless compatibility with existing electronic health record (EHR) systems is essential. This includes the secure transfer, storage, and interpretation of continuous data streams, which could enhance clinical decision-making but also raises concerns about data privacy and standardization. Overcoming these challenges will be essential to fully realize the potential of biosensors in delivering personalized, real-time care for patients with IBD.

## 8. Conclusions

Inflammatory Bowel Diseases are becoming more prevalent and represent a high burden on healthcare systems and the worldwide economy. They are debilitating diseases that cause great loss in patients’ quality of life. Its symptoms are usually misinterpreted, leading to lack of appropriate treatment and worst prognosis. Thus, there is a pressing need for the development of novel diagnostic tools that are specific and can be applied during each stage of the disease. However, the development of new diagnostic techniques is hampered by the lack of knowledge on specific biomarkers, mostly associated to the overlap of these markers with other pathologies. Nonetheless, some have been proven useful in different biological matrixes, namely sweat and feces, which is an advance in relation to more invasive sampling methods. This eventually can lead to more practical and widely used methods to help patients control the diseases.

The integration of biosensors into the realm of IBD diagnosis and prognosis represents a promising frontier in healthcare. The collective body of research showcased in this review underscores the potential of biosensors as invaluable tools, offering rapid, sensitive, and specific detection of biomarkers associated with IBD. These innovative technologies not only enhance the accuracy of early diagnosis but also contribute to ongoing disease monitoring, allowing for timely intervention and personalized treatment strategies. As we navigate the intricate landscape of IBD, biosensors emerge as indispensable allies, bridging the gap between traditional diagnostic methods and the evolving demands of precision medicine. Continued advancements in biosensor technology hold the key to transformative breakthroughs in the management of IBD, paving the way for more effective and patient-centric healthcare solutions.

## Figures and Tables

**Table 5:** Main genes involved in Inflammatory Bowel Disease (IBD) pathogenesis [100].

Role/Pathway	IBD	CD	UC
Innate mucosal defence	*CARD 9*, *RER*	*NOD2*, *ITLN1*	*SLC11A1*, *FGR2a/B*
Paneth cells	*XBP1*	*NOD2*, *ILTN1*, *ATG16L1*	
IL23/TH17	*IL23R*, *JAK2*, *TYK2*, *ICOSLG*, *TNFSF15*	*STAT3*	*IL21*
Autophagy	*CUL2*	*ATG16L1*, *IRGM*, *NOD2*, *LRRK2*	*PARK7*, *DAP*
Epithelial barrier		*MUC19*, *ILTN1*	*GNA12*, *HNF4A*, *CDH1*, *ERRFI1*
Immune cell recruitment	*MST1*	*CCL11*, *CCL2*, *CCL7*, *CCL8*, *CCR6*	*IL8RA*, *IL8RB*
T cell regulation	*TNFSF8*, *IL12B*, *IL23*, *PRDM1*, *ICOSLG*	*NDFIP1*, *TAGAP*, *IL2R*	*TNFRSF9*, *PIM3*, *IL/R*, *TNFSF8*, *IGNG*, *IL23*
Antigen presentation		*ERAP2*, *LNPEP*, *DENND1B*	
Immune tolerance	*IL10*, *CREM*	*IL27*, *SBNO2*, *NOD2*	*IL1R1*, *IL1R2*
Apoptosis	*PUS10*, *MST1*	*FASLG*, *THAA*	
ER stress	*ORMDL3*, *XBP1*	*CPEB4*	*SERNC3*
Oxidative stress	*CARD9*, *UTS2*, *PEX13*	*PRDX5*, *BACH2*, *ADO*, *GPC4*, *GPX1*, *SLC22A4*, *LRRK2*, *NOD2*	*HSPA6*, *DLD*, *PARK7*

**Table 6:** Genes with modified methylation profiles, reported in Inflammatory Bowel Diseases (IBDs) [100].

	Increased Methylation	Decreased Methylation
IBD vs. controls	*THRAP2*, *FANCC*, *GBGT1*, *WDR8*, *ITGB2*, *CARD9*, *CDH1*	*DOK2*, *TNFSF4*, *VMP1*, *ICAM3*
CD vs. UC	*CBGT1*, *IGFBP4*, *FAM10A4*	*IFITM1*
Active vs. remission	*CDH1*, *GDNF*, *SIT2*, *MDR1*, *FMR1*, *GXYLT2*, *RAFB*, *SLIT2*	*FOXA2*, *ROR1*, *NOTCH3*, *CDH17*, *PAD14*, *TNFSF8*, *EPHX1*, *HOXV2*, *FRK*
More aggressive vs. mild	*PAR2*, *MDR1*, *CDx1*, *RPS6KA2*	
Colorectal cancer risk	*RUNX3*, *MINT1*, *TGFB2*, *SLIT2*, *HS3ST2*, *TMEFF2*, *ITGA4*, *TFP12*, *FOXE1*, *SYNE1*, *APC*, *CDH13*, *MGMT*, *MLH1*, *nBMP3*, *NDRG4*, *EYA4*, *Vimentin*	*COX-2*

**Table 7:** miRNAs involved in Inflammatory Bowel Disease (IBD) [100].

	Increased Expression	Decreased Expression
IBD vs. controls	miRs-3180-3p, miRplus-E1035, miRplusF1159, miR20b, miR-98, miR125b-1 *, let-7e *, miRs-103-2 *, miR-362-3p, miR-532-3p, miR-98, miR340 *, miR-484	
Active vs. remission	miR-16, miR-21, miR-24, miR-126, miR-203, miR-28-5p, miR-151-5p, MiR-199a-5p, miR-340 *, miRplus-E1271, miR596, mir-199a-5p, miR-362-3p, miR-532-3p, miRplus-E1271, miR-877, miR-595	miR200b, mir-124, miRplus-F1075
More aggressive vs. mild	miR-29a, miR-29b, miR-29c, miR19a-3p, miR19b-3p, miR-31-5p	miR-196b-5p, miR-149-5p
Colorectal cancer risk	miR-31, miR-224, miR-21, miR-155, miR-26b, miR-15b, miR-17, miR-26b, miR-145	miR-143, miR-145

**Table 8:** Recent biosensing investigations showcasing various Inflammatory Bowel Disease (IBD) biomarkers and their respective LODs [115].

Biosensor Type	Material/Active Component	Marker/Detected Molecule	LOD	Reference
Electrochemical biosensor	VA-NCNT electrodes	lysozyme	100 fM	[116]
	Functionalized CNTs with amino groups	5-ASA and FA	36 and 3.1 nM, respectively	[117]
Electrochemical immunoassay	Iridium NP-loaded graphene	CRP	3.3 pg/mL	[118]
	Carbon electrode (SPE) loaded with AuNPs	CRP	0.15 nM	[114]
Endoscopy	Fecal immunochemical testing	fecal calprotectin (FCP); mucosal healing (MH)	100 ng/mL; 250 μg/g	[119]
FET sensor	Anti-TNF-α/CNT-SiO_2_	TNF-α	1 pg.L^−1^	[120]
Fiber optic-SPR bioassay	Functionalized Au-coated optical fibers	infliximab	2.2 ng/mL (15 pM)	[121]
Fluorescent sensor	P1–4/AgNC/cDNA probe	miRNA (miR-223)	0.018 μM	[122]
Immunosorbent assay	CdSe/ZnS QDs	CRP	0.46 ng/mL	[57]
Impedance spectroscopy based sensor	Polyamide/ZnO	CRP, IL-1β	0.2 pg/mL	[113]
Optical absorption spectroscopy	ML@PDDA	lysozyme	0.5 μg/mL	[123]
SERS quenching nanosensor	gold-coated copper oxide nanomaterial	TNF-α	173 pg/L	[124]
μQLIDA	PMMA microcapillary/MPO antibody/Quantum dots	myeloperoxidase	<5 nM	[125]
Waveguide-mode sensor	Streptavidin/AuNPs	CRP	10 pg/mL	[126]
Electrochemical Impedance Spectroscopy (EIS)	Wearable microneedle	CRP in interstitial fluid	0.7 µg/mL (buffer) and 0.8 µg/m	[127]
	Sweat-based EIS wearable	CRP, IL-6 andTNF-α in sweat	Distinction between inflamed and uninflamed state ~pg/mL ranges	[128]
	LIG-based electrochemical immunosensors	CRP and IL-6	1.45 pg/mL and 5.1 pg/mL, respectively	[129]
Microfluidic ELISA (mELISA)	ELISA microfluidic biosensor to quantify the therapeutic antibodies in IBD patient plasma samples	Anti-TNF-α on plasma	26 ng/mL (~6 times lower than standard ELISA)	[130]
Porous-silicon (Psi) Fabri-Pérot aptasensor	Gastrointestinal fluid	lactoferrin	1.8 μg/mL	[131]
CRISPR/Cas12a Colorimetric Assay	Portable tube-based CRISPR/Cas assay for point-of-care testing	Fecal calcoprotein	1 ng/ mL	[132]
Photonic Hydrogel sensor	Molecularly imprinted hydrogels and photonic crystals	Calprotectin on serum	70 pg/mL	[133]

**Table 9:** Analysis of the estimated costs, scalability, and practicality of the described biosensors according to the targeted biomarker.

Sensor Type/Platform	Target Biomarkers	Estimated Cost	Scalability	Practicality (Ease of Use)
Lateral Flow Assays (LFAs)	Fecal calprotectin	USD 5–30/test	High	Very High (home use)
Electrochemical Sensors (Aptamer/EIS)	CRP, IL-6, TNF-α	USD 10–50/device	Moderate-High	Moderate (requires reader)
Wearable Microneedle/EIS Sensors	CRP, IL-6	USD 100–300/device	Low	Moderate (needs calibration)
Lab-on-Chip ELISA (Microfluidic)	Cytokines, calprotectin, mAbs	USD 50–100/test	Moderate	Moderate (some user steps)
FO-SPR/SPR Platforms	Infliximab, adalimumab	USD 200–1000/setup	Low	Low (lab-based, skilled use)
Nucleic Acid Biochips	miRNA, bacterial DNA (oral/gut)	USD 50–200/test	Low-Moderate	Low (pre-processing needed)
CRISPR-based Colorimetric Assays	Fecal calprotectin	~USD 5–10/test	Moderate-High	High (minimal training)
Wearable Saliva Sensors/Oral Patches	Oral microbiome, IL-1β, IL-6	USD 50–150/device	Low	Low-Moderate (conceptual phase)

## Data Availability

No new data were generated or analyzed in support of this research.

## Abbreviations

5-ASA	aminosalycilate drug mesalazine
ASCAs	Anti-Saccharomyces cerevisae Antibodies
AuNPs	Gold Nanoparticles
CAI	Colitis Activity Index
CARD15	Caspase Recruitment Domain family member 15
CD	Crohn’s Disease
CDAI	Crohn’s Disease Activity Index
CLE	Confocal Laser Endomicroscopy
CNT	Carbon Nanotubes
CP	Calprotectin
CRP	C-reactive Protein
CT	Computed Tomography
DBE	Double-Ballon Assisted Endoscopy
ELISA	Enzyme-Linked Immunosorbent Assay
ERS	Erythrocyte Sedimentation Rate
FA	Folic acid
FET	Field-Effect Transistor
GWASs	Genome-wide Association Studies
HLA	Human Leukocyte Complex
IBD	Inflammatory Bowel Disease
IBS	Irritable Bowel Syndrome
IL-1β	interleukin-1β
IL23-R	Interleukin-24 receptor
LCN-2	Lipocalin-2
LOD	Limit of Detection
LRG	Leucine-rich alpha 2-glycoprotein
miRNAs	MicroRNAs
ML	Micrococcus lysodeikticus
MRE	Magnetic Resonance Enterography
NMs	Nanomaterials
NOD2	Nucleotide-binding Oligomerization Domain-containing protein 2
NPs	Nanoparticles
p-ANCAs	Perinuclear Anti-neutrophil Cytoplasmic Antibodies
PDDA	poly(diallyldimethylamonium)
PGE-MUM	Prostaglandin E-major
SBE	Single-Ballon Assisted Endoscopy
SERS	Surface-Enhanced Raman spectroscopy
SNPs	Single-Nucleotide Polymorphisms
SPE	Screen-Printed carbon Electrode
SPR	Surface Plasmon Resonance
SWV	Square Wave Voltammetry
TNF-α	Tumor Necrosis Factor-alpha
UC	Ulcerative Colitis
US	Ultrasound
VA-NCNT	Vertically aligned nitrogen-doped carbon nanotube.
VCE	Video Capsule Endoscopy
μQLIDA	Quantum Dot-Linked Immuno-Diagnostic Assay
